# Effects of ketamine on individual symptoms and symptom networks of depression in a randomised controlled trial of ketamine for treatment-resistant depression

**DOI:** 10.1192/bjp.2024.276

**Published:** 2025-11

**Authors:** Shabnam Hossein, Manivel Rengasamy, Aiyedun Uzamere, Crystal Spotts, Robert H. Howland, Meredith L. Wallace, Sanjay J. Mathew, Rebecca B. Price

**Affiliations:** Department of Psychiatry, University of Pittsburgh School of Medicine, Pittsburgh, Pennsylvania, USA; Department of Psychology, University of Pittsburgh, Pittsburgh, Pennsylvania, USA; Department of Psychiatry, Baylor College of Medicine, Houston, Texas, USA

**Keywords:** Biological, depression, ketamine, out-patient treatment, randomised controlled trial

## Abstract

**Background:**

Understanding the effects of ketamine on depressive symptoms could help identify which patients might benefit and clarify its mechanism of action in both the early (≤1 day post-infusion) and late (e.g. 2–30 days post-infusion) post-infusion periods. Symptom network analyses could provide complementary information regarding relationships between symptoms.

**Aims:**

To identify the effects of ketamine on symptom-level changes in depression across both the early and late post-infusion periods and on depressive symptom network changes.

**Methods:**

In this secondary analysis of 152 adults with treatment-resistant depression (with 38.8% reporting suicidal ideation at baseline), we compared symptom changes in the early and late post-infusion periods between individuals randomised to a single 40 min infusion of intravenous ketamine 0.5 mg/kg (*n* = 103) or saline (*n* = 49) and identified changes in symptom networks between pre- and post-ketamine treatment using network analyses.

**Results:**

In the early post-infusion period, the greatest improvement (comparing ketamine with saline) was in depressive symptoms related to sadness. In network analyses, symptom network connectivity increased following ketamine infusion. Symptoms of sadness and lassitude showed persistent improvement in the first week post-infusion, whereas improvements in suicidal thoughts first emerged 3–4 weeks post-infusion.

**Conclusion:**

Ketamine improved all symptoms but showed the greatest effect on symptoms of sadness, both immediately and in the initial week after treatment. Ketamine also rapidly altered the topology of symptom networks, strengthening interrelationships between residual symptoms. The efficacy of ketamine (compared with saline) regarding suicidal symptoms emerged later. Our findings suggest potentially divergent efficacy, time courses and mechanisms for different symptoms of depression.

In recent years, ketamine has emerged as an effective rapid-acting treatment for depression and treatment-resistant depression (TRD).^1^ Intravenous (i.v.) ketamine treatment seems to have maximal effects approximately 24 h after infusion, with persistence of effects for 1–2 weeks after treatment, and approximately a 40–46% response rate in this timeframe.^2,3^ Although many patients experience temporary reduction in overall depression severity following a single infusion of ketamine, high symptom heterogeneity in depression may contribute to divergent outcomes and differential efficacy for individual symptoms in particular timeframes (e.g. more rapid-acting versus slower-acting effects).^4^ An understanding of the differential effects of ketamine on individual depressive symptoms could help guide both treatment decisions and future mechanistic research.

Early open-label studies explored the effects of ketamine on individual symptoms of depression and initially suggested that ketamine more prominently affected suicidal thoughts^5,6^ or anhedonia.^7^ However, a recent pooled analysis of three randomised controlled trials (RCTs) examining the effects of ketamine within the first 3 days of treatment of unipolar or bipolar depression suggested that ketamine most robustly improved sadness, pessimistic thoughts, concentration difficulty and numbness.^8^ These mixed findings suggest a lack of clarity with respect to which symptoms ketamine might most robustly affect in individuals with unipolar TRD – who represent the majority of patients currently receiving i.v. ketamine in real-world clinical practice. Few studies in individuals with TRD have examined individual symptom changes that are specific to ketamine, e.g. by including a placebo arm that clarifies effects unique to ketamine.^8^ Importantly, a specific need to understand the effects of ketamine on suicidal ideation and/or behaviours has been emphasised in recent ketamine-related research, given that this is a time-sensitive, urgent clinical need in patients with depression and can also be an index of overall distress associated with morbidity and mortality.^9^

## Rationale for study

Furthermore, both durability of symptom improvements and late-emerging effects on symptoms following a single infusion of i.v. ketamine remain understudied.^10,11^ Although the global effects of ketamine on depression severity are broadly thought to peak in the first day after infusion and gradually reduce after 1–2 weeks post-infusion, the time course of efficacy at the individual symptom level is relatively unknown.^3^ These effects are important given that certain patients (e.g. acutely suicidal patients) may require careful clinical management in the early post-infusion period if ketamine does not rapidly target specific symptoms prominently endorsed by those patients. Other patients might more comfortably space the follow-up treatments (e.g. repeated ketamine doses) if their prominent symptoms are improved in a more enduring or slower-acting pattern. This could have significant logistical, financial and safety benefits for patients and the healthcare system. Early research suggests that in addition to clinical efficacy, mechanisms of improvement may differ between the early post-infusion period following ketamine treatment (e.g. ≤1 day; where ketamine has been shown to have the greatest efficacy) and the late post-infusion period following ketamine infusion (e.g. >1 day to 30 days).^12–14^ Clarity regarding the timeframe of symptom-level changes would provide both further elucidation of the mechanistic nuances of ketamine sequelae and valuable clinical knowledge necessary to calibrate the timing of treatment administration.

Importantly, symptoms of depression do not change independently of each other but rather covary with each other and affect one another (e.g. improving insomnia can improve fatigue). Symptom networks^15,16^ are a statistical tool that can be used to examine this complex interplay among depression symptoms. Symptom networks can complement more traditional statistical methods by enabling focus on the patterns of pairwise relationships present between depression symptoms. They allow visualisation of statistical relations, which can help to identify how relationships among depressive symptoms change as a result of treatment. Recently, an innovative technique called network intervention analysis (NIA) has extended traditional network analysis by using more advanced statistical techniques to identify which symptoms in a symptom network might be related to direct (rather than indirect) effects of treatment (e.g. determining whether treatment first improves mood, the improvements in which are then associated with subsequent changes in suicidal thoughts).^15,17^ Thus, NIA could be used to distinguish between direct and indirect effects related to ketamine (relative to placebo) treatment, providing insight for researchers exploring mechanisms of ketamine and also identifying symptom profiles of patients who might benefit from both the direct and indirect effects of ketamine.

## Study hypotheses and objectives

To help address the gaps in the literature, we performed a secondary analysis to describe and examine the effects of a single-dose of i.v. ketamine on individual symptoms of depression in a double-blind RCT in a cohort of participants with unipolar TRD who received a single infusion of ketamine or saline (*N* = 152; *n*_saline_ = 49; *n*_ketamine_ = 103). To our knowledge, this constituted the largest RCT analysis to date examining symptom-level effects of i.v. ketamine. First, we examined the effect sizes for improvements in specific symptoms between the ketamine and placebo arms in the early post-infusion period (i.e. ≤1 day post-infusion), where peak antidepressant effects were observed. Second, we assessed individual symptom improvement related to ketamine in the later treatment period (i.e. post-infusion days 2–30), examining individual symptom profiles over time, with a specific focus on the temporal profile of the effects of ketamine (relative to saline) on suicidal ideation. On the basis of the literature, we hypothesised that the effects of ketamine on suicidal ideation would be most prominent in the early treatment period. Finally, given the lack of prior literature examining symptom network changes related to ketamine, we sought to clarify how ketamine would change the associations between individual symptoms of depression, including determination of which specific symptoms of depression were affected most directly by ketamine in our sample.

## Methods

Data for the current secondary analyses was obtained from a previously published clinical trial (R01MH113857; ClinicalTrials.gov: NCT03237286).^18^ Findings described previously for the parent trial focused on overall depression severity and did not include any analysis of specific symptoms.

### Participants

Participants included in analyses were 152 individuals with TRD (mean age, 34.3 years; 62.5% female; 81.5% Caucasian) randomised to a single dose of i.v. ketamine (*n* = 103; 0.5 mg/kg over 40 min) or i.v. saline placebo (*n* = 49; 50 mL 0.9% NaCl) and who completed, at a minimum, baseline and 24 h clinical assessments (see CONSORT flowchart in the Supplementary material, available online at https://doi.org/10.1192/bjp.2024.276). Inclusion criteria included history of nonresponse to one or more FDA-approved antidepressant medications, Montgomery–Åsberg Depression Rating Scale (MADRS) score ≥25, stable psychiatric medications for 4 weeks before the baseline assessment (if taking psychiatric medications) and low self-worth based on self-report measures (see Price et al^18^ for further description). Exclusion criteria included lifetime history of mania or psychosis, current substance or alcohol use disorder, pregnancy or serious unstable medical illnesses. Study participants were recruited from the community and out-patient clinical settings through website listings, clinical referrals and a local research registry. Further details and full inclusion and exclusion criteria are available at ClinicalTrials.gov: NCT03237286. Additional details of the study characteristics and procedures are described in the previously published parent trial.^18^ Written consent was obtained from all participants. The authors assert that all procedures contributing to this work comply with the ethical standards of the relevant national and institutional committees on human experimentation and with the Helsinki Declaration of 1975, as revised in 2013. All procedures involving human subjects and/or patients were approved by the University of Pittsburgh Institutional Review Board (no. 19040414). Participants were first recruited for the study on 1 December 2017.

### Clinical measures

Current analyses focused primarily on MADRS symptom scores (which ranged from 0 to 6) across ten different MADRS items, with higher symptom scores indicating greater symptom severity.^19^ MADRS symptom score ratings at all time points (pre-infusion baseline, post-infusion days 1, 5, 12, 21 and 30) were assigned by a clinical rater. A secondary outcome measure was the Columbia-Suicide Severity Rating Scale (C-SSRS), which includes a five-item binary ‘suicidal ideation’ scale assessing presence or absence of different levels of suicidal ideation.^20^ For the present analyses, we defined active suicidal ideation as a response of ‘yes’ to question 2 of the C-SSRS suicidal ideation section (capturing any endorsement of active suicidal ideation). Of note, patients endorsing suicidal intent (‘yes’ response to question 4 and/or question 5 on the C-SSRS) at any study time point were excluded from the study and referred for a higher level of care.

### Statistical analysis

We used R statistical software (version 4.2.1) to conduct statistical analyses; details of the analyses are described in Supplementary Material 2. Given our study design and recent meta-analyses of the antidepressant effects of ketamine suggesting that maximal effects of ketamine appear approximately 24 h post-infusion, we separated our analyses into an ‘early’ post-infusion period (baseline to 24 h) and ‘late’ post-infusion period (days 5 to 30).^18^ The early period included pre-infusion baseline and post-infusion day 1 assessment of depression symptoms. Immediately following the day 1 assessments, participants entered the late post-infusion period, in which they received either a cognitive training intervention or sham cognitive intervention (see Supplementary Material 2 for further details and an explanation of analytic decisions related to these groups).^21^ Given our previously published findings suggesting that the cognitive training intervention uniquely increased the durability of the effects of ketamine on overall depression severity,^18,22^ patients in the ketamine + cognitive training arm (*n* = 53) were excluded from the present late analyses to ensure our detected effects of ketamine on individual symptoms were conservative, more likely to be reproducible (including in archival ketamine study data-sets) and generalisable to real clinical settings (in which ketamine is currently provided without this adjunctive treatment). To maximise sample sizes and enable reliable network estimation, network analyses were conducted only for the early post-infusion period (which included 103 participants). Fifty-three participants received the cognitive training treatment between 2 and 5 days post-infusion, which affected depression trajectories in the late post-infusion period, and were thus excluded from these analyses (as noted above; see also Supplementary Material 1); therefore, we were unable to reliably estimate networks for the remaining participants included in analyses in the late post-infusion period (*n* = 50).

#### Missing data

For analysis of the early post-infusion period, data were fully available for all participants (*n* = 103). For analysis of the late post-infusion period, multiple imputation (using R package *mice* and function *mice*, *m* = 50 copies of the data-set) was used to impute missing data (7.7% of events).^24^

### Early post-infusion period analysis

#### Symptom change analysis

We calculated the difference in effect size between the ketamine and saline cohorts (Cohen’s *d*) for each individual MADRS item score at 1 day post-infusion. Raw symptom scores at each time point (as opposed to change scores) were used to enhance clinical meaningfulness and reduce potential statistical errors related to floor effects or baseline measurement variance.

#### Network analysis

Network estimation and visualisation: symptom networks were estimated by using a Gaussian graphical model. In the symptom network models, nodes represented individual depressive symptoms (measured by MADRS items), and an edge between two nodes represented a conditional dependence relation (partial correlation), that is, the correlation between two symptoms after controlling for the influence of all the other symptoms. The networks were visualised using the *qgraph* package (see Supplementary Material 2). The accuracy and stability of the networks are described in Supplementary Material 3.

Network comparison: differences between networks at baseline and 24 h post-infusion were compared using the *NetworkComparisonTest* R package. We also tested for changes in the network structure, individual edges and global network strength. Global strength, which was defined as the sum of the edge weights of a network, is a measure of how strongly the symptoms are connected in the network. Median *P*-values are reported from 100 independent executions of *NetworkComparisonTest* (see Supplementary Material 2).

Network intervention analysis: we used mixed Gaussian graphical models to estimate networks consisting of depression symptom nodes and a treatment condition (ketamine versus saline) node at baseline and 24 h post-infusion (see Supplementary Material 2 and 3 for further details). In this context, mixed Gaussian graphical models enable identification of which symptoms might be conditionally dependent on the treatment variable, potentially suggesting which symptoms are most directly affected by the treatment variable. However, care should be used when interpreting the results of NIA, which are not inferential and thus may not necessarily be generalisable beyond the analysed sample.

### Late post-infusion period analyses

#### Symptom change analysis

As in the early post-infusion analyses, we calculated the effect size difference between the ketamine and saline cohorts (i.e. Cohen’s *d*) for each individual MADRS symptom at a given post-infusion time point (post-infusion day 5, 12, 21 or 30).

#### Restricted mean survival time (RMST) analysis

To analyse differences between the ketamine and saline groups in terms of recurrence of suicidal ideation in the late post-infusion period (using our outcome measure of the C-SSRS), we used RMST analysis, which enables identification of the total area under a survival curve and is considered to be a more reliable estimate than mean survival times.^23^ RMST effectively identifies differences in cumulative probability of an event occurring between two groups over a set period of time (see Supplementary Material 2 for details). We also conducted logistic regression analyses to obtain odds ratios relating prediction of active suicidal ideation (using the suicidal ideation dummy variable) by cohort at each given time point.

## Results

### Baseline clinical characteristics

At baseline, for both treatment groups, the mean score on each item exceeded the MADRS scale’s midpoint (score >3) for most items, with the exception of the reduced appetite and suicide ideation items (Table 1). At baseline, 25.7% of all participants endorsed active suicidal ideation on the C-SSRS, while 38.8% of participants endorsed any type of suicidal ideation (defined as answering ‘yes’ to any item on the five-item ‘suicidal ideation’ scale on the C-SSRS or MADRS SI item score >1).

**Table 1:** Demographic and clinical variables at pre-infusion baseline

Demographic and clinical variables	Early post-infusion period cohort^a^				Late post-infusion period cohort^b^			
	Ketamine	Saline	Effect size (Cohen’s *d* or phi)	*P*-value^d^	Ketamine (without ASAT)^c^	Saline	Effect size (Cohen’s *d* or phi)	*P*-value^d^
	*n* = 103	*n* = 49			*n* = 50	*n* = 49		
Variable	Baseline, mean/percentage				Baseline mean/percentage			
Age (mean, years)	34.631	33.551	0.102	0.549	34.560	33.551	0.092	0.646
Biological sex (% male)	37.864	36.735	0.011	0.893	36.000	36.735	−0.008	0.939
Race/ethnicity (% Non-Hispanic Caucasian)	82.353	79.592	0.033	0.893	81.633	79.592	0.026	0.939
Number of failed antidepressant trials	2.631	2.735	−0.053	0.791	2.600	2.735	−0.064	0.752
MADRS symptom	Mean baseline symptom score				Mean baseline symptom score			
Reported sadness	3.99	3.939	0.077	0.648	3.980	3.939	0.058	0.775
Apparent sadness	3.932	3.918	0.021	0.901	3.900	3.918	−0.027	0.892
Inner tension	3.359	3.184	0.158	0.393	3.380	3.184	0.166	0.41
Reduced sleep	3.476	3.633	−0.13	0.407	3.720	3.633	0.089	0.659
Reduced appetite	1.602	1.959	−0.204	0.25	1.780	1.959	−0.101	0.617
Concentration difficulties	3.709	3.265	0.391	0.049	3.740	3.265	0.375	0.066
Lassitude	3.699	3.673	0.023	0.896	3.700	3.673	0.025	0.901
Inability to feel	3.786	3.673	0.119	0.489	3.800	3.673	0.127	0.529
Pessimistic thoughts	3.728	3.612	0.097	0.563	3.760	3.612	0.12	0.551
Suicidal thoughts	1.583	1.592	−0.006	0.973	1.720	1.592	0.083	0.681
C-SSRS^e^	Percentage				Percentage			
Endorsing passive death wish (baseline)	34.951	46.939	−0.115	0.215	38.000	46.939	−0.09	0.486
Endorsing active suicidal ideation (baseline)	24.272	28.571	−0.046	0.712	26.000	28.571	−0.029	0.951

MADRS, Montgomery–Åsberg Depression Rating Scale.

a. The full ketamine-treated cohort included in early post-infusion period analyses.

b. The Smaller ketamine-treated cohort included in the late post-infusion period analyses.

c. ASAT (automated self-association training) refers to the cognitive training intervention conducted in a subset of ketamine participants (excluded from analyses of the late post-infusion period, which focused on the stand-alone effects of ketamine).

d. *P*-values reflect differences between groups based on *t*-tests (continuous variables) or chi-squared tests (categorical variables).

e. For Columbia-Suicide Severity Rating Scale (C-SSRS) analyses, passive death wish was defined as a response of ‘yes’ to question 1 on the C-SSRS, whereas active suicidal ideation was defined as a response of ‘yes’ to question 2 on the C-SSRS.

### Early post-infusion period: symptom changes

**Fig. 1:**
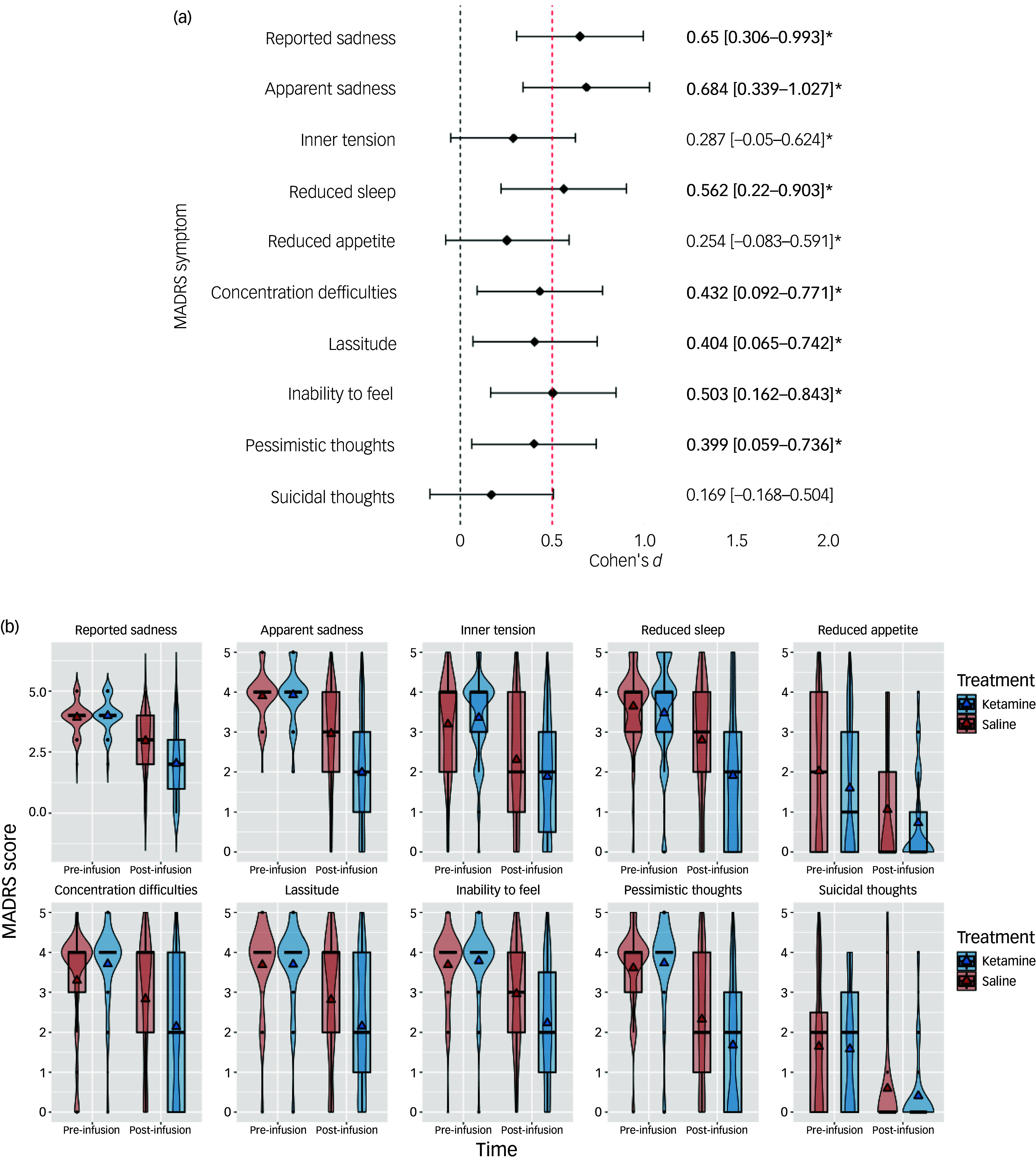
Early post-infusion period symptom differences. (a) Cohen’s *d* values comparing differences in Montgomery–Åsberg Depression Rating Scale (MADRS) symptom scores between the saline (*n* = 49) and ketamine (*n* = 103) cohorts 1 day after infusion, with 95% confidence intervals. Asterisks and bold text indicate statistically significant differences in symptom scores between the ketamine and saline cohorts. Dotted reference lines indicating no effect (Cohen’s *d* = 0) and medium effect (Cohen’s *d* = 0.5) are provided for ease of interpretability. (b) Distributions of ten MADRS symptoms pre-infusion (left half of each plot) and 24 h post-infusion (right half of each plot). The box plots visualise MADRS symptom values of the first, second (median) and third quartiles, and the superimposed violin plots depict the distribution of the symptom values. Triangles represent the mean values of symptom scores.

When participants receiving ketamine (*n* = 103) were compared with those receiving saline placebo (*n* = 49), the greatest effects of ketamine at 1 day post-infusion were on symptoms of apparent sadness and reported sadness (Cohen’s *d* ≥ 0.65, *P* < 0.05). Statistically significant medium-to-large differences were also observed for pessimistic thoughts, inability to feel, concentration difficulties, lassitude and reduced sleep (Cohen’s *d* ≥ 0.39, *P* < 0.05) (Fig. 1). No between-group differences were found with respect to suicidal thoughts reported on the MADRS (Cohen’s *d* = 0.169, *P* = 0.38).

### Early post-infusion period: network analyses

#### Network estimation and stability

**Fig. 2:**
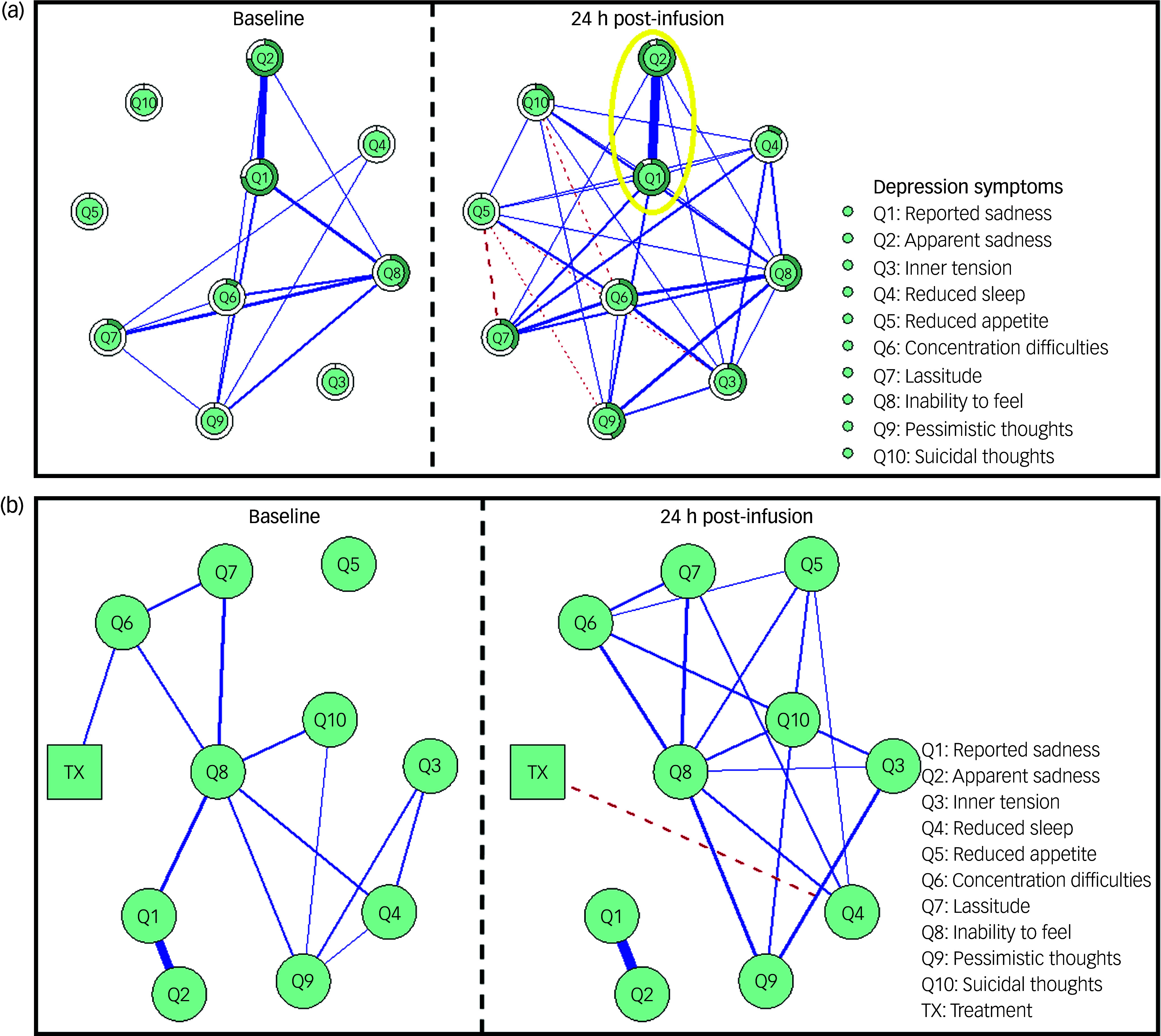
Symptom network changes related to ketamine treatment. (a) Networks displaying the relationship between depressive symptoms as measured by Montgomery–Åsberg Depression Rating Scale at baseline and at 24 h post-infusion. Blue lines indicate positive associations, dashed grey lines indicate negative associations, and thickness and brightness of an edge represent association strength. Rings around nodes show predictability, with shadowed parts depicting variance explained by connected nodes. Overall, a larger number of visualised edges shown at 24 h post-infusion reflects a significant increase in the global strength of the network from pre- to post-infusion (*P*_median_ < 0.001). The black circle marks the specific edge weight that significantly increased from pre-infusion to 24 h post-infusion. (b) Regularised mixed Gaussian graphical model networks estimated pre-infusion and 24 h post-infusion from the network intervention analysis, including treatment as a node. Circular nodes indicate items from the Montgomery–Åsberg Depression Rating Scale, and the square node indicates treatment (1, ketamine; 0, saline). Edges between symptoms nodes represent conditional dependence relations or unique associations among variables controlling for all the other variables in the network. Edges are parameterised as regression coefficients from generalised linear regression models. Thus, edges between symptoms can be interpreted to be similar to partial correlations, and edges between the treatment node and symptoms as regression coefficients. Positive edges are depicted with blue lines and negative edges with grey dotted lines. The positive edge between the treatment node and concentration difficulties was due to the initial difference between treatment groups for this symptom (see also Table 1). At 24 h, a negative edge between the treatment node and sleep symptom is visible. This suggests that in the current sample, ketamine directly improved sleep symptoms more than saline at 24 h, whereas the effects of ketamine on other symptoms might be more indirect. The results of this analysis are considered to be descriptive rather than inferential and thus may not necessarily generalise beyond the analysed sample.

In both the pre-infusion and 24 h post-infusion networks (Fig. 2a), the edges between reported sadness and apparent sadness had the greatest weights (0.61 and 0.88, respectively). Detailed results are presented in Supplementary Material 3.

#### Network comparison: baseline versus 24 h post-ketamine

Network comparison permutation tests showed that the networks did not significantly differ in terms of their overall network structure (*P*_median_ > 0.39); however, the global strength of the network was significantly higher post-infusion than pre-infusion (*P*_median_ < 0.001), suggesting that symptoms were more densely connected 24 h post-ketamine. With respect to specific edge weights, after correction for multiple comparisons using the false discovery rate, we found a significant increase in the weight of the edge between reported sadness and apparent sadness from pre- to post-ketamine infusion (*P*_median_ = 0.049).

#### Network intervention analysis

The NIA results suggested that reduced sleep was the symptom most directly affected by ketamine 24 h post-infusion in our sample, whereas other symptoms may have changed as a result of more indirect effects (through other symptoms); see Fig. 2b and Supplementary Material 3.

### Late post-infusion symptom changes: symptom change

**Fig. 3:**
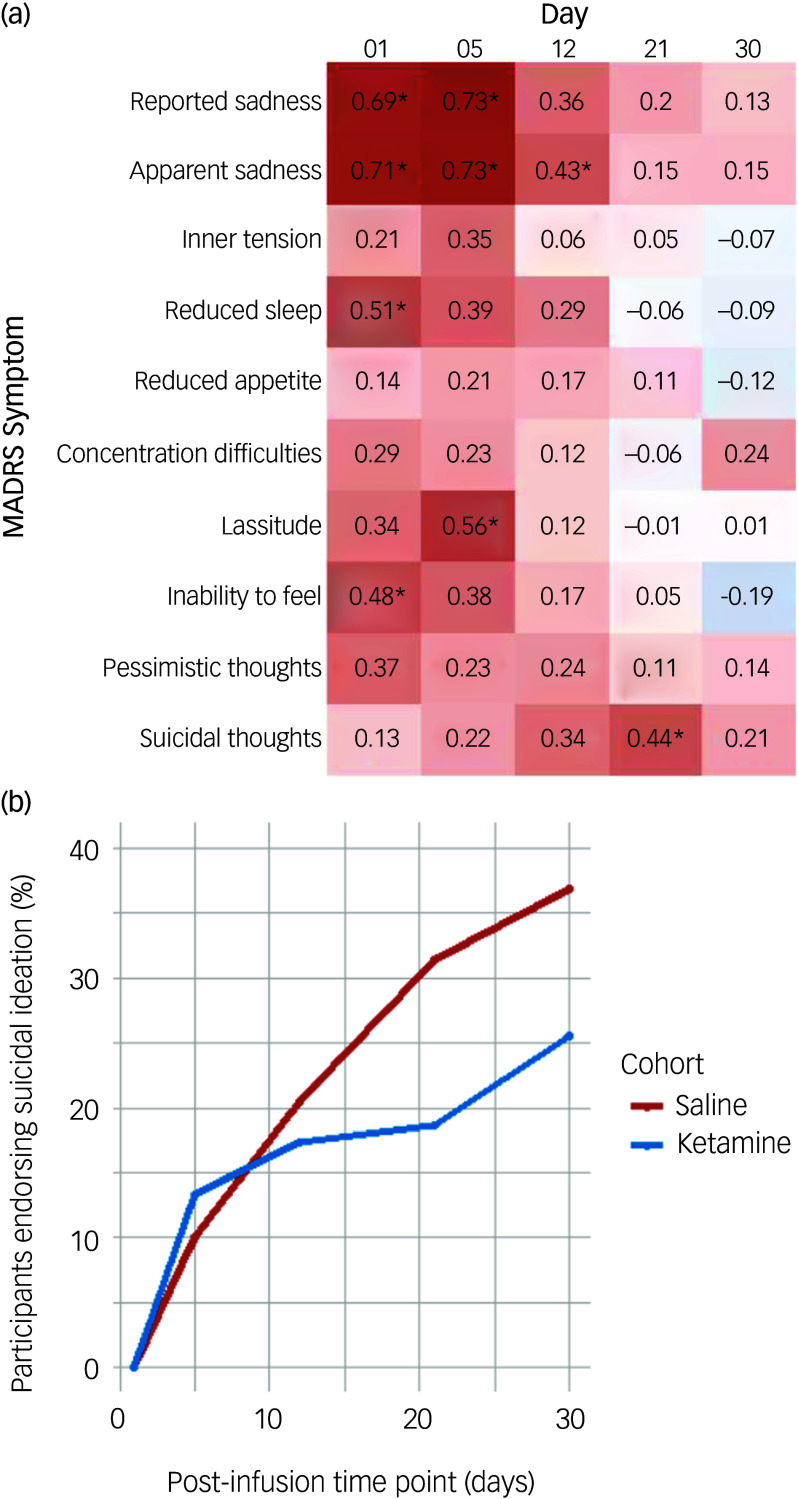
Late post-infusion period symptom differences. (a) Table illustrating Cohen’s *d* values comparing Montgomery–Åsberg Depression Rating Scale (MADRS) symptom scores between ketamine and saline groups at the given time point, for analyses comparing the saline (*n* = 49) and ketamine (*n* = 50) cohorts. For descriptive purposes, Cohen’s *d* for day 1 is also presented for the present ketamine-only subsample. Asterisks indicate a significant difference. (b) Descriptive figure of percentages of recurrence of active suicidal ideation, corresponding to restricted mean survival time analyses. Data shown are cumulative percentages of participants who endorsed an event of active suicidal ideation in the specified study period (between each time point and post-infusion day 1) among individuals who did not endorse suicidal ideation at post-infusion day 1, for both the ketamine (*n* = 47) and saline (*n* = 42) cohorts. Because the analysis includes only individuals who were free of suicidal ideation at post-infusion day 1, the cumulative percentage starts at 0% on that day and increases over time as recurrences occur.

For individuals receiving ketamine as a standalone treatment (*n* = 50) compared with those receiving saline (*n* = 49), significant improvements in symptoms persisted to post-infusion day 5 for apparent sadness, reported sadness and lassitude (Cohen’s *d* > 0.56, *P* < 0.05) and to day 12 for apparent sadness only (Cohen’s *d* = 0.43, *P* < 0.05) (Fig. 3a). No other statistically significant group differences were observed for individual symptom items from the MADRS during the late post-infusion stage, with one exception: for suicidal thoughts, the greatest differential improvement in symptoms (ketamine > saline) was found later in the post-infusion period on post-infusion day 21 (Cohen’s *d* = 0.44, *P* < 0.05), but no statistically significant group differences in suicidal thoughts were found at any other time point (either before or after day 21). See Supplementary Material 4 for effect sizes of within-group symptom improvements.

### Recurrence of suicidal ideation

When examining individuals who endorsed no active suicidal ideation at post-infusion day 1 based on the C-SSRS (*n*_ketamine_ = 47, *n*_saline_ = 42), ketamine (compared with saline) led to significant and greater reductions in cumulative recurrence of suicidal ideation based on RMST analysis (Fig. 3b), specifically at post-infusion day 30 (estimate = 2.02, *P* = 0.01) but not at earlier post-infusion time points (*P* > 0.12). Whereas RMST analysis was used to examine differences in time to risk of recurrence of suicidal ideation, logistic regression analyses provided information on differences in odds of suicidal ideation endorsement at a given time point, including statistically lower odds of endorsed active suicidal ideation in ketamine participants at post-infusion day 21 (odds ratio = 0.24, *P* = 0.023) and lower (but not statistically significantly different) odds at other late-period time points (day 12: odds ratio = 0.64, *P* = 0.45; day 30: odds ratio = 0.58, *P* = 0.32).

## Discussion

In this study, which was among the largest RCTs of single-dose infusion of i.v. ketamine to date, we examined changes in individual symptoms of depression and how these varied at different time points in the post-infusion period (e.g. up to 30 days post-infusion), comparing active ketamine treatment with saline placebo. Our findings suggest that in contrast to prior open-label trials examining symptom level changes, ketamine has the greatest impact on symptoms related to sadness immediately after treatment, whereas effects on suicidal ideation (relative to placebo) may peak after a greater delay. We have also described several novel effects of ketamine on the interrelations between individual symptoms of depression (symptom networks), based on the first analysis (to our knowledge) examining the effects of ketamine on symptom networks.

Our findings in the early post-infusion period, based on a placebo-controlled RCT design, suggested a specific early profile of ketamine-related symptom improvement (notably, improvements in sadness, pessimistic thoughts and inability to sleep; Fig. 1) and lesser or no differential improvements (relative to saline) in suicidal thoughts, reduced appetite or anxiety (inner tension). Such findings are remarkably similar to those of a prior cross-over pooled RCT analysis, which suggested that early post-infusion period improvements were most prominent for symptoms of pessimistic thoughts, hypersomnia, inability to feel and lassitude, with lesser improvements in appetite and suicidal thoughts.^8^ Conjunctively, these findings suggest that ketamine-specific improvements, particularly improvements in mood, pessimistic thoughts and sleep, may be related to rapid-acting biological mechanisms proposed by prior researchers, such as effects on synaptic growth (e.g. in the hippocampus via factors such as brain-derived neurotrophic factor^25^), neuroplasticity, changes in neurotransmitter activity or alterations in neural networks. Such findings will better allow researchers to focus on why particular mechanisms pertain to specific clinical symptom improvements and also identify patients that might benefit more from the rapid effects of ketamine.

The results of our symptom network analysis add to the literature on the relationship between symptom network topology and depression treatment;^26–29^ mixed results have been reported by studies relating network connectivity to clinically relevant topics such as psychopathology severity, risk, treatment response prediction and the effects of treatment.^27–30^ Our findings suggest that ketamine increased the global strength of the partial correlation symptom network. Given robust treatment response rates overall in the present sample, higher symptom network connectivity may be reflective of greater interrelationships between residual symptoms, which had lower variance compared with pre-infusion symptoms. Future work could directly compare different treatments to determine whether the impact of ketamine on symptom networks differs from that of other treatments (e.g. slower-acting conventional medications). In terms of edge weight changes, the higher edge weight between reported and apparent sadness at baseline and the increase in this edge weight after ketamine infusion are concordant with our findings showing that these symptoms specifically improve the most with ketamine treatment and are closely related to each other, becoming even more tightly intertwined following ketamine treatment. In addition, our NIA results suggested that reduced sleep was the symptom most directly affected by ketamine 24 h post-infusion in the present sample. This is in line with prior literature suggesting that the rapid antidepressant effects of i.v. ketamine may be partially mediated by improved sleep as measured by self-report and objective sleep markers; however, this finding requires replication.^31–33^

In the late post-infusion period, the only symptom that showed persistent improvements greater than placebo past post-infusion day 5 was apparent (observed) sadness (for which improvements persisted to day 12 but not beyond); this indicates a clinical need to deploy serial ketamine infusions in fairly quick succession to maintain rapid gains, as is commonly done in clinical practice,^2,34^ and/or to deploy adjunctive treatments with the potential to extend the effects of ketamine.^18^ This strategy is probably indicated across a wide range of heterogeneous clinical presentations of TRD, as we did not identify any subset of symptoms that showed reliable, longer-lasting effects above and beyond those of placebo.

Regarding the effects of ketamine on suicidal ideation within a relatively low-risk sample (i.e. no active suicidal intent at baseline or recent or ongoing suicidal crises), our findings suggest that the timeframe of its peak effects on suicidal ideation (days 21–30 post-infusion) may lag compared with that of its peak effects on depressive symptoms (days 1–5 post-infusion) with the effects on suicidal ideation becoming stronger after significant effects on depression severity were no longer detected. These findings may be related to the protective effects among the saline group of non-specific factors (e.g. frequent interactions with research staff) and timing-related factors (e.g. less opportunity for discrete instances of suicidal ideation to be captured by research instruments when querying about a very brief interval), which appeared to have a waning influence over the course of the 30-day period (e.g. see Supplementary Material 4, suicidal thoughts item, for waning within-group effects in the saline arm), allowing ketamine-specific beneficial impacts to emerge.^35^ Late-period improvements in suicidal ideation could also be linked to earlier improvements in other symptoms of depression (e.g. sleep). Our findings extend those of smaller preliminary studies of esketamine and/or ketamine studies that suggested minimal impact of ketamine on suicidal ideation in the early post-infusion period and/or a greater impact in the late post-infusion period (compared with placebo).^9,10,36^ Given potential floor effects in our study (e.g. if baseline suicidal ideation scores had been higher, rapid differences between ketamine and saline might have been detected), future RCTs examining patients with higher baseline levels of suicidal ideation would be beneficial; only ∼25% of participants in our study endorsed any active suicidal ideation at baseline, which may explain the divergence of our findings from those of a prior meta-analysis that identified effects of ketamine on suicidality in the early post-infusion period.^6^ Our findings are also in contrast to those of a meta-analysis which found minimal late post-infusion effects on suicidal ideation following a single dose of ketamine;^37^ this suggests that variability across clinical populations and/or study designs is likely to affect the durability of effects on suicidal ideation. Broadly, our findings suggest that the mechanism underlying the effects of ketamine on suicidal ideation and/or behaviour may be partially dissociable from its effects on other depressive symptoms and potentially tied to more slowly developing processes such as effects on neural networks or downstream effects on receptor regulation;^38,39^ thus, our results generate testable hypotheses for further research.

Our study had some limitations. The aims of the present analyses were exploratory and hypothesis-generating; thus, we did not adjust for multiple comparisons in most analyses. This minimised the risk of type II errors but may have increased the risk of type I errors. Our saline placebo group also received a digital cognitive intervention at post-infusion days 2–5, which may have led to underestimation of ketamine versus placebo effect sizes in late post-infusion analyses. Network analyses are limited in that associations observed between nodes are correlational rather than causal; the NIA (unlike the other network analyses we conducted) is considered to be descriptive rather than inferential and thus may not be generalisable beyond the present sample; and the sample size in the saline arm did not permit reliable estimation of independent network characteristics within this arm. In addition, the current trial was not designed with the goal of delineating the impact of ketamine on suicidal ideation *per se*. Thus, patients at higher risk of suicidal thoughts and behaviours were excluded from the study and referred for a higher level of care, and assessments of suicidal ideation relied solely on individual items from the MADRS or C-SSRS rather than more comprehensive assessments. Although prior studies have reported similar results using extensive questionnaires to assess suicidal ideation and/or behaviour,^37^ several ongoing trials will be better equipped to inform clinically relevant conclusions regarding the rapid and/or enduring effects of ketamine on suicidal ideation and behaviour among higher-risk cohorts. Finally, although treatment arms were very well matched on a wide range of clinical and demographic factors, including overall depression severity and overall level of treatment resistance, between-group differences were observed in a single baseline symptom (concentration; Table 1); this suggests a failure of randomisation to generate identical samples on this specific symptom measure, which may have affected results involving this symptom.

Of note, given that our findings suggest the potential for multiple unique mechanisms of change in recovery, future studies might benefit from using longer follow-up periods and repeated doses of ketamine. For instance, one theoretical explanation for our early post-infusion findings (i.e. stronger connectivity of depressive symptoms in the post-infusion period) is that the brain is focused on recovery at this time, enabling more bottom-up or automatic, internal regulation of co-occurring symptoms; however, further research is needed to replicate and validate plausible interpretations of this finding. Directly comparison of ketamine with other conventional antidepressant treatments could help to identify mechanisms specific to ketamine that affect symptoms of depression. Last, examining other timeframes of symptom change which have largely been understudied in the existing literature (e.g. 12 or 36 h post-infusion) or identifying more fine-grained timeframes of change could provide valuable insight into the effects of ketamine on individual symptoms of depression.

In conclusion, in this relatively large RCT of ketamine (versus saline) of adults with TRD, we found that treatment with ketamine was characterised by greater differential impacts on specific symptoms of depression (notably improvements in sadness) along with global increases in connectivity between symptoms in the early post-infusion period. Our NIA results also suggested that the most direct effect of ketamine within this sample was on improved sleep, with effects on all other symptoms possibly lying downstream (as indirect effects). In the late post-infusion period (21–30 days post-infusion), improvements in suicidal ideation in the ketamine group (over and above those in the saline group) emerged which were not observed in the earlier post-infusion period. Our findings overall suggest that within the heterogeneous clinical presentation of depression, specific symptom profiles are more responsive to a single infusion of ketamine than others, and that specific symptom effects can be either early and transient or later-emerging. Findings encourage future research to investigate greater individualisation of ketamine treatment and explore distinct mechanisms that may characterise differing profiles of symptom improvement over time.

## Supporting information

Hossein et al. supplementary material Hossein et al. supplementary material

## Data Availability

The data, analytic code and materials supporting the findings of this study are available from the corresponding author, M.R., upon reasonable request.
