# Supplementary material for: Effects of ketamine on individual symptoms and symptom networks of depression in a randomised controlled trial of ketamine for treatment-resistant depression
Source: Br J Psychiatry. 2025 May 13;227(5):756–65. doi: 10.1192/bjp.2024.276 (PMC12310196; doi:10.1192/bjp.2024.276)
Supplement: Hossein et al. supplementary material [file S0007125024002769sup001.docx]

**Supplemental Material**

**Table of Contents**

Supplement S1. Consort Flowchart

Supplement S2. Methodological Details Related to Analyses

Supplement S3. Network Analyses Details

Supplement S3A. Network Accuracy and Stability – Network Comparison Test

Supplement S3B. Network Accuracy and Stability – Network Intervention Analysis

Supplement S4. Raw Symptom Score Change Effect Size

**Supplement S1.** Consort Flowchart. Diagram adapted from Price et al., 2022 [1]. ASAT refers to automated self-association training (cognitive training intervention), with further description in the Methods.


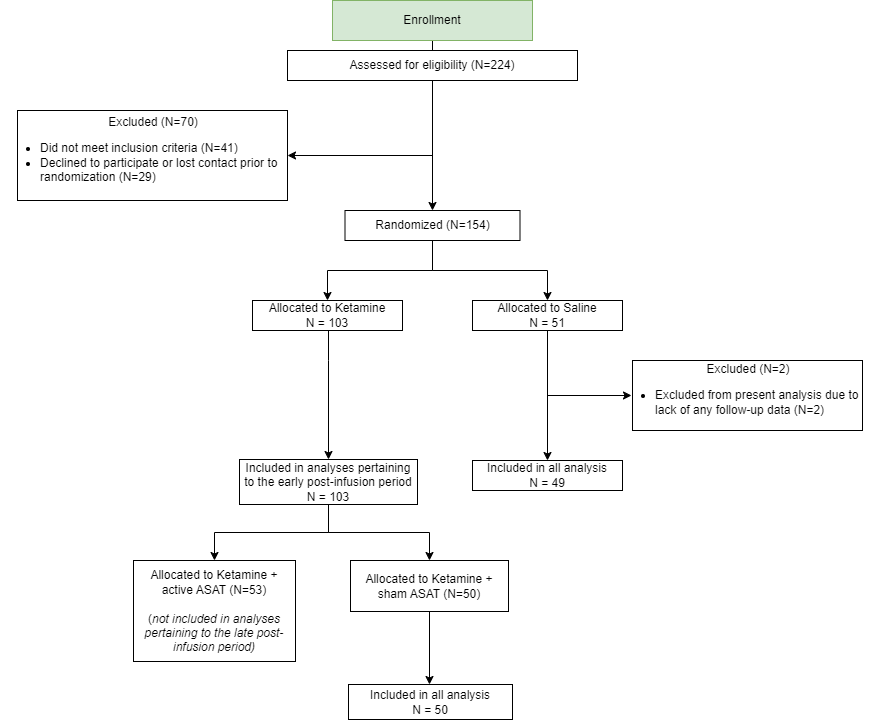


**Supplement S2.** Methodological Details Related to Analyses.

**R Packages Utilized: for Network Analyses.** Network analyses using *bootnet* R package (v1.5.6; Epskamp et al., 2018), *qgraph* R package (v1.9.8; Epskamp et al., 2012), *mgm* R package (v1.2-14; Haslbeck & Waldorp, 2020), and *NetworkComparisonTest* R package (v2.2.2; C. D. van Borkulo et al., 2023).

**Network Estimation.** LASSO shrinks all coefficients towards zero and sets small weights exactly to zero. The strength of the shrinkage is controlled via a tuning parameter, which is selected by minimizing the Extended Bayesian Criterion (EBIC). EBIC also has a hyperparameter, that controls to what extent the EBIC favors simpler models with fewer edges. For both of these parameters the default values of *estimateNetwork* function in the *bootnet* package were used (gamma = 0.5, lambda.min.ratio = 0.01). To increase specificity and reduce the risk of overfitting, the GGMs were estimated by using the least absolute shrinkage and selection operator (LASSO).

**Network Visualization.** Each node corresponds to a MADRS item. The strength of the association between two nodes is represented by the thickness of the edge between them. Positive associations are shown with blue and negative associations with red edges. “Predictability”, estimating how well a node can be predicted by the other nodes of the network, was calculated with *mgm* package, and shown as rings around nodes, with shadowed parts depicting variance explained by the connected nodes. The layout was fixed for all the networks to allow for visual comparison between their structures. The accuracy and stability of the estimated networks are reported in the Supplement (see Supplemental Table of Contents).

**Network Comparison.** We compared networks using the *NetworkComparisonTest* R package (v2.2.2; NCT; C. D. van Borkulo et al., 2023). NCT is a permutation test algorithm. It assesses the difference between two networks based on several invariance measures (network structure, global strength, and edge invariance). To ensure robustness of results, we executed the NCT permutation algorithm 100 times, using 10,000 iterations (permutations) within each algorithm execution. This methodology was used given expected slight variations in p values between different executions dependent on the random seed used to generate random numbers used in the permutation test algorithm in the software *R*. For this reason, median p values (derived from 100 executions of the NCT algorithm which resulted in 100 p values) are reported in the primary text, consistent with methodologies in prior studies[2]. To maximize sample sizes and allow for reliable network estimation, network comparison tests focused primarily on the ketamine arm (n=103).

**Network Intervention Analysis.** For NIAs, MGMs are used to depict pairwise association between nodes that are of different types of variables (e.g., ordinal and categorical). *mgm* R package (v1.2-14; Haslbeck & Waldorp, 2020) was used to compute these networks. Since the *mgm* function in the *mgm* package handles only categorical and continuous variables, MADRS scores were considered continuous. Similar to the GGMs, LASSO was used to regularize models. The data-driven cross-validation approach with ten folds in this function was used to determine the regularization hyperparameters (Bernstein et al., 2023; Blanken et al., 2019; Curtiss et al., 2021). To maximize sample sizes and allow for reliable network estimation, Network Intervention Analysis included participants in both arms (n=152).

**RMST Analysis.** RMST presents effects in terms of the time to event difference between groups over a given period of time. To allow for quantifying time-to-recurrence of active SI via RMST, we examined only participants who denied active suicidal thoughts (as per the C-SSRS; answering “No” to Q2 - Q5 on the SI portion of the C-SSRS) at post-infusion Day 1 (n_ketamine_=47; n_saline_=42). We then used the R package *survRM2 v1.0-4* (*rmst2* function) to calculate the cumulative difference between ketamine and saline groups of recurrence of active SI over time (using a “SI dummy variable” identifying if the participant expressed active SI or not at a given timepoint)[3].

**Exclusion of Participants in the ketamine+cognitive training arm from late period analyses.** Of note, the reference group for our late period analyses (saline+cognitive training) received the active cognitive intervention; although there was no clear indication that the cognitive training intervention produced any beneficial stand-alone impacts on symptoms (when paired with saline), in the event that it did, the present late-period analyses would therefore yield a conservative estimate of ketamine’s effects at later timepoints.

**Supplement S3. Network Analyses Details**

**Supplement S3A. Network accuracy and stability of networks analyzed in Network Comparison Test.** Pre-infusion and post-infusion networks accuracy and stability metrics are presented. Stability of the estimated edges of the network and their accuracy were calculated using bootstrapping methods. First using nonparametric bootstrapping, the 95% confidence intervals of the edge weights were calculated using the *bootnet* function in the *bootnet* package (1000 bootstrap samples)(Epskamp et al., 2018) indicating their accuracy. To assess the stability of the edge weights, the correlation stability coefficient (CS) was calculated using case-dropping bootstrap with the same *bootnet* function. The CS coefficient identifies the maximum proportion of cases that can be dropped from the data while still retaining, with 95% probability, a correlation of at least 0.7 (default) between edge weights calculated based on the original data and edge weights computed with less cases. A CS coefficient of greater than 0.50 is preferred. The correlation stability (CS) coefficients for the edge weights of both networks were greater than 0.5 (0.592, and 0.67 respectively) suggesting good stability.

Figure Legend. The bootstrapped confidence intervals of the estimated edge weights for the estimated network of 10 MADRS symptoms at each time point. The red line indicates the sample values. The gray areas indicate the bootstrapped confidence intervals. Each horizontal line represents one edge of the network, ordered from the edge with the largest edge weight to the edge with the smallest edge weight. The *y*-axis labels indicate the edges.

**Supplement S3B. Network Accuracy and Stability of networks analyzed in Network Intervention Analysis.** Pre-infusion and post-infusion networks accuracy and stability metrics are presented, with treatment cohort included as a node in the network. Stability of the estimated edges of the networks and their accuracy were calculated using bootstrapping methods. First using nonparametric bootstrapping, the 95% confidence intervals of the edge weights were calculated using the *bootnet* function in the *bootnet* package (1000 bootstrap samples)(Epskamp et al., 2018) indicating their accuracy. To assess the stability of the edge weights, the correlation stability coefficient (CS) was calculated using case-dropping bootstrap with the same *bootnet* function. The CS coefficient identifies the maximum proportion of cases that can be dropped from the data while still retaining, with 95% probability, a correlation of at least 0.7 (default) between edge weights calculated based on the original data and edge weights computed with less cases. A CS coefficient of greater than 0.50 is preferred. The correlation stability (CS) coefficients for the edge weights of both networks were greater than 0.5 (CS = 0.75) suggesting good stability.

| Pre-infusion Network | Post-infusion Network |
| --- | --- |
|  | |

Figure Legend. The bootstrapped confidence intervals of the estimated edge weights for the estimated network of 10 MADRS symptoms at each time point. The red line indicates the sample values. The gray areas indicate the bootstrapped confidence intervals. Each horizontal line represents one edge of the network, ordered from the edge with the largest edge weight to the edge with the smallest edge weight. The *y*-axis labels indicate the edges.

**Supplement S4.** Raw Symptom Score Change Effect Size. These tables detail Cohen’s D of raw symptom score differences across different timepoints (post-infusion Day 1, 5, 12, 21, or 30) as compared to the baseline timepoint for either the ketamine (n=50) or saline group (n=49). To calculate raw symptom score differences, the mean raw difference between MADRS symptom scores at given timepoint (e.g., post-infusion Day 5) and at the baseline timepoint was computed. Cohen’s D of the raw symptom score differences are presented for the ketamine and saline, as per existing statistical recommendations[4]. A negative Cohen’s D value indicates an effect size reflecting a decrease in symptom score at the given from baseline. The findings here generally suggested large raw symptom changes and moderate to large effect size differences in MADRS symptom changes in both ketamine and saline groups, with greater raw symptom changes and effect size differences earlier in the post-infusion period for ketamine participants.

Table 1. Within-groups Cohen’s D comparing MADRS symptom scores at a given timepoint (Days 1, 5, 12, 21, or 30) compared to baseline MADRS symptom scores within the ketamine and saline cohorts.

| **Ketamine** | **Saline** |
| --- | --- |
| 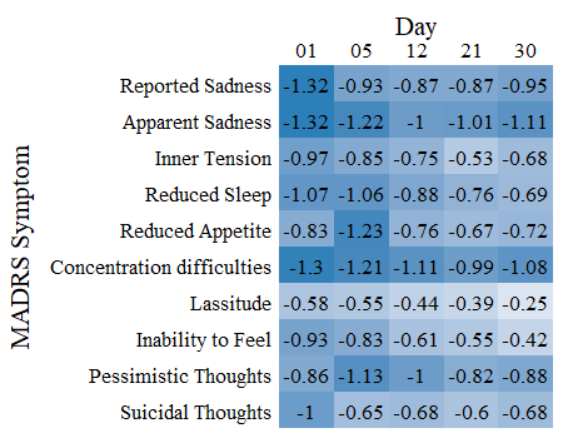 | 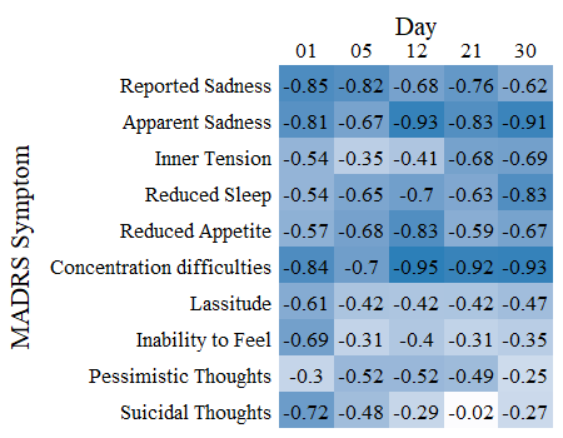 |

Supplemental References

1. Price, R.B., et al., *A novel, brief, fully automated intervention to extend the antidepressant effect of a single ketamine infusion: a randomized clinical trial.* American Journal of Psychiatry, 2022. **179**(12): p. 959-968.

2. Weintraub, M.J., C.D. Schneck, and D.J. Miklowitz, *Network analysis of mood symptoms in adolescents with or at high risk for bipolar disorder.* Bipolar disorders, 2020. **22**(2): p. 128-138.

3. Uno, H., et al., *survRM2: Comparing restricted mean survival time.* R package version, 2020: p. 1.0-3.

4. Dankel, S.J. and J.P. Loenneke, *Effect sizes for paired data should use the change score variability rather than the pre-test variability.* The Journal of Strength & Conditioning Research, 2021. **35**(6): p. 1773-1778.
